# The South African Society of Psychiatrists’ response to the White Paper for National Health Insurance in South Africa

**DOI:** 10.4102/sajpsychiatry.v22i1.1053

**Published:** 2016-10-26

**Authors:** Lesley J. Robertson

**Affiliations:** 1 Department of Psychiatry, University of the Witwatersrand, South Africa

The South African Department of Health (DoH) released its White Paper for National Health Insurance (NHI) in December 2015 for public comment. The South African Society of Psychiatrists (SASOP) submitted a detailed response following discussion amongst both public and private sector members. This has been published as a pdf in the August 2016 edition of *South African Psychiatry*, available at www.southafricanpsychiatry.co.za. For the most part, our response was concerned with the proposed organisation of health services as they relate to mental health care. However, we also made comment on good governance within the health service and on human rights issues. In this respect, we responded as advocates for our patients, for the public good and for our profession. Essentially, we submitted a response as participants in the proposed transformation of the health system appropriate to our social contract with society.

## The social contract

The social contract is described as an unwritten agreement between the medical profession and society. The latter includes government and health care funders on the one hand and patients and the public on the other hand.^1^ Each body has implied expectations of and obligations towards the other in a three-way relationship. In responding to the invitation for comment by the DoH on NHI, we satisfied the following expectations by government of the medical profession, as outlined by Cruess and Cruess:^1^

Participation in ‘team health care’ in terms of policy development
Being a ‘source of objective advice’ within our field of expertise
Providing active ‘promotion of the public good’.

In turn, we exercised our own expectation to play a ‘role in developing health policy’. Within our response, we have emphasised the need for our participation as psychiatrists in the NHI Benefits Advisory Committee and the National Essential Medicines List (NEML). This is vital if we need to have professional autonomy in clinical decision-making. We also touched on the need for parity between the public and private sectors in terms of working conditions and remuneration. We made the most detailed comment, however, on a major omission of the NHI White Paper, that is, the lack of provision for any community-based psychiatry where the majority of the mentally ill should be able to access care. In so doing, we asserted our constitutionally sanctioned expectation of government to provide an ‘adequately funded and staffed health care system’ in which we may optimally practice and teach our profession. As this expectation is shared by the public, inclusive of good and suitable quality of care, our response is an expression of mental health advocacy and our social contract with our patients.

## Mental health advocacy

Most low- and middle-income countries (LMICs) reportedly spend a meagre percentage of their health budget allocated to mental illness on specialised hospitals rather than community-based care, despite the cost-effectiveness of community care and even when a mental health policy exists.^2^ The NHI White Paper reveals South Africa to be no exception. No psychiatric care is provided for up to regional hospital level, in contradiction to the precept for accessible community-based care of the *Mental Health Care Act* of 2002 and the National Mental Health Policy Framework and Strategic Plan 2013–2020. Although there is provision for primary mental health care, it is without specialist support. In addition, mental health is not included in community or school outreach programmes. Point 199 of the NHI White Paper goes so far as to state that ‘Specialised Psychiatric Services are services that may be provided in general hospitals … but are mostly provided at specialised facilities designed for the care of mentally ill patients’.^3^

Through conducting qualitative interviews with mental health leaders and experts, Saraceno et al. elucidated several causes for the low priority given to mental health, particularly community-based care, in LMICs.^4^ Of note are the competing public health priority conditions, a misinterpretation of the 1978 Alma Alta Declaration as if to mean that primary mental health care alone may provide ‘health for all’, a lack of public mental health leadership and weak, inconsistent and sometimes conflictual mental health advocacy by both professionals and the public. They call for a collaborative, clear voice by mental health professionals to advocate for improved mental health coverage through decentralised quality care. Importantly, the voice and the language used to describe the mental health issues must be easily understood by health authorities and pertinent to the general health agenda.

We hope that we achieved this goal in our chapter-by-chapter commentary on the White Paper. To maintain simplicity, we narrowed our focus to the need for specialist-run community-based psychiatry. We proposed a model for community mental health teams that would provide supervision and support to primary mental health care, specialist community-based mental health assessments, facilitation of patient referrals and engagement with the non-health sector with respect to mental health promotion. Comments on other aspects of service provision such as budgetary concerns and the NEML were made in light of community care. Hence, although we may not have documented the needs of all SASOP members, it is hoped that our submission on the White Paper expressed the most pertinent of our expectations of government, addressing its and our obligations towards the mentally ill in the society.
